# Respiratory depression in women receiving propofol/esketamine versus propofol/fentanyl for abortion surgery or curettage: a randomized clinical trial

**DOI:** 10.1080/07853890.2025.2483981

**Published:** 2025-04-02

**Authors:** Cuiwen Zhang, Jiaxin Luo, Yeqing Liao, Huiyu Cao, Xiaoshan Wu, Xiaofang Huang, Hongmeng Lan, Yuliu Lin, Huihe Chen, Xuehai Guan

**Affiliations:** ^a^ Department of Anaesthesiology, The First Affiliated Hospital of Guangxi Medical University, Nanning, PR China; ^b^ Department of Rehabilitation, The First Affiliated Hospital of Guangxi Medical University, Nanning, PR China

**Keywords:** Abortion surgery, clinical trial, curettage, esketamine, fentanyl, respiratory depression

## Abstract

**Background:**

A combination of opioids with propofol is a popular approach to non-intubated general anaesthesia; however, this method usually results in higher incidence of respiratory depression. We compared the incidence of esketamine- and fentanyl-induced respiratory depression in women undergoing abortion surgery or curettage under propofol-based non-intubated general anaesthesia.

**Methods:**

This study included 176 women (aged 18–60 years) scheduled for abortion surgery or curettage. Patients were randomized into the fentanyl or esketamine groups. Patients in the fentanyl group received fentanyl (1 µg/kg) combined with propofol intravenously. Patients in the esketamine group received subanaesthetic doses of esketamine (0.15 mg/kg) combined with propofol intravenously. The primary outcome was the incidence of respiratory depression during anaesthesia. Secondary outcomes included respiratory rate, pulse oximetry, blood pressure, heart rate, propofol dose, duration of surgery, duration of anaesthesia, and adverse events.

**Results:**

The incidence of respiratory depression in the esketamine group was significantly lower than that in the fentanyl group (11% vs. 45%; *p* < .0001). Propofol administration was lower with esketamine than fentanyl. Respiratory rate, SpO_2_ and blood pressure were more stable in the esketamine group than in the fentanyl group. The incidences of hypotension, propofol-induced injection pain and chin lifting in the esketamine group were lower than those in the fentanyl group. The incidence of nightmares was higher in the esketamine than in the fentanyl group.

**Conclusions:**

The incidence of respiratory depression was lower with subanaesthetic doses of esketamine than with fentanyl in women undergoing abortion surgery or curettage under propofol-based non-intubated general anaesthesia.

**KEY MESSAGES**
- Opioids combined with propofol is a popular method for non-intubated general anaesthesia; however, this method usually results in higher incidence of respiratory depression.
- At subanaesthetic doses, esketamine provides an analgesic effect by antagonizing the N-methyl-d-aspartate receptor.
- In this trial, the incidence of respiratory depression was lower with subanaesthetic doses of esketamine than with fentanyl in women undergoing abortion surgery or curettage under propofol-based non-intubated general anaesthesia.

## Introduction

Respiratory depression is a complication of anaesthesia with serious short- and long-term consequences for patients, and can lead to fatalities. Respiratory rate, depth and rhythm are the three essential elements of respiratory ventilation. Consequently, attention should be paid to respiratory depression during anaesthesia.

Propofol is a popular sedative used for general anaesthesia. Propofol acts on γ-aminobutyric acid (GABA) A receptors, mediates neuronal hyperpolarization and induces loss of consciousness [1]. It is widely used for general anaesthesia due to its rapid onset, rapid offset and low incidence of associated nausea and vomiting. However, propofol can cause cardiorespiratory depression, involving both central respiratory nucleus suppression and airway impairment [2,3]. Propofol-induced respiratory depression (PIRD) occurs mainly through a central mechanism, resulting in low genioglossus muscle activity [4]. Propofol reduces the activity of pre-inspiratory neurons, resulting in decreasing of the inspiratory burst rate [5]. PIRD is partly caused by activation of GABA receptors in the pre-Bötzinger complex, which is the kernel of respiratory rhythmogenesis [6]. Clinical doses of propofol during anaesthesia induction lead to a 25–30% incidence of apnoea [7]. Propofol can reduce tidal volume, decrease respiratory rate dose-dependently, and result in unpredictable changes in minute volumes [8,9]. Indeed, respiratory depression involving decreased tidal and minute volumes and reduced ventilatory response to hypoxia is the most severe adverse event associated with propofol use in clinical settings [9,10].

Opioids are effective analgesics; however, they have a number of serious drawbacks such as tolerance development, itching, urinary retention, addiction and life-threatening respiratory depression, which limit their efficacy in clinical practice [11,12]. Respiratory depression is also the primary problem associated with opioid use or abuse [13]. Opioid-induced respiratory depression (OIRD), specifically a decrease in tidal volume and respiratory rate, may result in dysrhythmias, cardiac ischemia, cardiac arrest and even death. By activating μ-opioid receptors expressed on neurons in the respiratory circuits within the brainstem, opioids depress chemosensory respiratory responses, causing respiratory depression [14–16]. Inhibition of hypercapnic and hypoxic chemosensory reflexes results in an inadequate response to decreased PaO_2_ and increasing PaCO_2_ levels during hypoventilation [17,18]. Tidal volumes are reduced with increasing doses of opioids, with decreased phrenic motor neuron activity [19]. Opioids also increase breathing variability, even at low doses [20]. Opioids inhibit the upper airways, resulting in obstructive sleep apnoea, swallowing difficulty and aspiration, even when using doses that only mildly suppress the respiratory rate [21]. Respiratory muscles also become rigid with exposure to opioids, including morphine and heroin, and are particularly common with fentanyl [22]. This muscle rigidity further impairs ventilation and reduces tidal volume.

Abortion surgery and curettage are common procedures in gynaecological outpatient surgery centres. Using a combination of propofol with fentanyl for non-intubated general anaesthesia provides pain relief for patients undergoing abortion surgery or curettage. However, as the use of propofol or fentanyl alone may each induce respiratory depression, a combination of propofol with fentanyl for non-intubated general anaesthesia may further worsen the situation.

One strategy for combating OIRD is the development of alternative analgesics with minimal respiratory depressive effects [23]. Ketamine, a mixture of esketamine and levo-ketamine, is a narcotic sedative and analgesic drug that does not affect respiration [24,25]. By blocking the connection between the thalamus and the limbic system, ketamine induces a dissociative anaesthetic state at anaesthetic doses [26]. The use of ketamine alone as an analgesic or sedative is limited due to its sympathetic effects, associated involuntary physical movements, and frequent nausea and vomiting [27,28]. The analgesic effect and affinity of esketamine for the N-methyl-d-aspartate receptor (NMDAR) are twice those of ketamine, and it causes few adverse events [29,30]. At subanaesthetic doses, esketamine provides an analgesic effect by antagonizing the NMDAR [31].

We hypothesized that the incidence of respiratory depression with subanaesthetic doses of esketamine as analgesic in patients undergoing abortion surgery or curettage under propofol-based non-intubated general anaesthesia would be lower than with fentanyl. The aim of the study was to compare the incidence of respiratory depression induced by esketamine and fentanyl in women undergoing abortion surgery or curettage under propofol based non-intubated general anaesthesia.

## Materials and methods

### Study design and patient enrolment

This was a single-centre, double-blind, randomized clinical trial. The trial was conducted at the First Affiliated Hospital of Guangxi Medical University. The trial protocol was approved by the Medical Ethics Committee of the First Affiliated Hospital of Guangxi Medical University (No. 2022-KY-E-045) and was registered at Chictr.org.cn (Identifier: ChiCTR2200057602) on 15 March 2022. Patients were enrolled between 18 March 2022 and 31 December 2022. Written informed consent was obtained before enrolment. No significant changes to the protocol occurred after the trial commenced. This study adhered to the Consolidated Standards of Reporting Trials guidelines (Supplement material) and the tenets of the Declaration of Helsinki.

We enrolled female patients (aged 18–60 years, American Society of Anesthesiologists (ASA) physical status I–II) undergoing abortion surgery or curettage under general anaesthesia. Exclusion criteria included patients with a history of liver dysfunction, kidney dysfunction, neurological diseases (such as epilepsy, psychosis or increased intracranial pressure), cardiovascular diseases (such as cardiac dysfunction (NYHA class III or IV), systolic blood pressure (SBP) ≥180 mmHg, diastolic blood pressure (DBP) ≥110 mmHg, cor pulmonale or pulmonary hypertension), allergy to the drugs under study, opioid abuse, pathological obesity (body mass index (BMI) ≥30 kg/m^2^), difficult airway, increased intraocular pressure, or hyperthyroidism.

### Randomization and group allocation

Random numbers were generated by using EpiCalc 2000 software (Joe Gilman and Mark myatt) in a 1:1 ratio. Randomization numbers were concealed using sequentially numbered opaque envelopes, which were retained by a trial coordinator. Before anaesthesia, the trial coordinator, who did not take part in the study, opened the envelope indicating group assignment, and passed this information to the attending anaesthesiologist (X.H.G.) who then used previously prepared identically appearing esketamine or fentanyl–atropine mixtures as required.

Patients, attending anaesthesiologists, surgeons, other health care members, and investigators responsible for recruitment, data collection and analysis were blinded to the group allocation. Unmasking was allowed only in case of an emergency, such as a life-threatening event.

### Anaesthesia management and intervention

All patients fasted for 6 h prior to the procedure. No premedications were administered. A 22-gauge cannula was inserted into the dorsal vein of the patient’s hand. Ringer’s lactate solution (5 ml/kg/h) was infused to maintain patency. In the operating room, respiratory rate, electrocardiography and pulse oximetry (SpO_2_) were monitored continuously, and SBP and DBP were measured using a cyclic model during anaesthesia induction, followed by every 3 min during anaesthesia maintenance. Oxygen was administered at an initial rate of 2 L/min (standard flow rate at our institution) via a double nasal catheter. During anaesthesia induction, patients in the fentanyl group received a mixture of fentanyl (Humanwell, Yichang, China) and atropine (1 μg/kg and 5 μg/kg, respectively, as regularly used at our institution), followed by propofol (1.5 mg/kg, with an additional dose of 0.5 mg/kg each time at 3 min intervals until the Modified Observer’s Assessment Alertness/Sedation Scale [MOAA/S] reached 0; JiaBo, Guangzhou, China). Patients in the esketamine group received esketamine (0.15 mg/kg; Hengrui, Lianyungang, China), followed by propofol as described above until MOAA/S reached 0. During anaesthesia maintenance, propofol was administered as the surgery progressed in both groups. All drugs were stopped at the end of the surgery.

### Outcome parameters

The primary outcome of the study was the incidence of respiratory depression during the anaesthesia, which was measured using one of the following standards: respiratory rate ≤8 breaths/min measured by capnography, apnoea ≥15 s, or SpO_2_ ≤ 90% (≥15 s). If SpO_2_ ≤90% continued for more than 15 s, the following sequence of steps would be used to corrected hypoxemia: increasing the oxygen flow rate, lifting the patient’s chin, inserting an oropharyngeal airway, and providing artificial assisted ventilation. Secondary outcomes included SBP, DBP, heart rate, respiratory rate, SpO_2_, propofol dose and duration of anaesthesia and surgery. Adverse events such as hypertension (≥20% increase in SBP from baseline), hypotension (≥20% decrease in SBP from baseline), propofol-induced pain (PIP, assessed by a four-point pain score), bradycardia (<50 beats/min), tachycardia (>100 beats/min), need for chin lifting, physical movement, cough, nausea/vomiting, pleasant dreams, nightmares, headache and dizziness were also recorded. Blood pressure five minutes before anaesthesia was defined as the baseline blood pressure. All adverse events were evaluated from the time of anaesthesia induction to 1 h after surgery. Dreams were evaluated by 1 h after surgery and were defined according to the principle of happiness. Dreaming of a happy relationship, mastering wealth and status, having powerful abilities, buying a desirable house, and so on, was defined as pleasant dreams. Dreams that left the patient feeling uncomfortable, stressed or anxious were defined as nightmares.

### Sample size calculation

Our preliminary trial showed that the incidences of respiratory depression were 35% and 16% in the fentanyl and esketamine groups, respectively. The sample size was calculated using PASS software (version 11.0; NCSS, Kaysville, UT), assumed a type-I error of 0.05. Eighty samples were required in each group to allow detection of a difference between groups (procedure: two independent proportions power analysis; two-sided, *Z* test with pooled variance; sample allocation ratio = 1; *α* = 0.05, *β* = 80%). Considering a potential dropout rate of 10%, the sample size of each group was increased to 88.

### Statistical analysis

Outcome analysis was performed in the intention-to-treat (ITT) population. For the primary outcomes, after excluding those with protocol deviations, an analysis was performed on the per-protocol (PP) population.

The normality of data distribution in continuous variables was tested by using the Kolmogorov–Smirnov test, followed by the *F*-test for equality. Continuous variables with a normal distribution and equal variance are presented as means (standard deviations [SDs]), and were compared between groups by using the unpaired *t*-test. Repeated-measures data, such as SBP, DBP, mean blood pressure (MBP), heart rate, SpO_2_ and respiratory rate were compared using repeated-measures two-way analysis of variance with Greenhouse–Geisser’s correction, followed by Bonferroni’s multiple comparison test. Continuous variables with non-normal distribution or with unequal variance are presented as medians (interquartile ranges (IQRs)), and were compared between groups by using the Mann–Whitney *U*-test. The difference in medians (95% confidence intervals (CIs)) was also calculated. Categorical variables are showed as numbers (%) and were compared between groups by using Fisher’s exact test. The primary outcomes and adverse events were analysed using Fisher’s exact test, and the relative risk and 95% CI were also calculated. Significance was assumed at a two-sided *p* < .05.

Data were analysed using GraphPad Prism 9.0 (Dotmatics, San Diego, CA).

## Results

**Figure 1.**
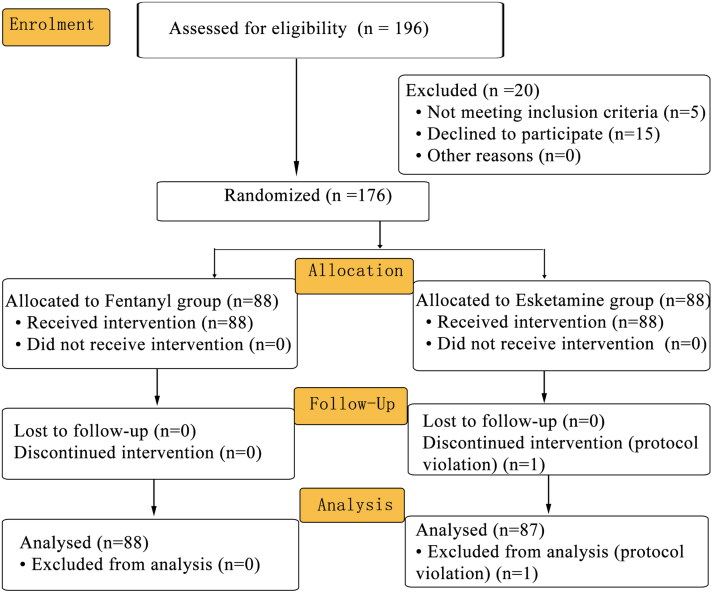
CONSORT diagram for the trial. CONSORT: Consolidated Standards for Reporting Trials.

Of 196 patients who were assessed for eligibility, 176 patients (mean [SD] age, 29.9 [6.4] years; mean [SD] height, 159.8 [9.2] cm; mean [SD] weight, 53.3 [8.1] kg; mean [SD] BMI, 20.9 [3.1] kg.m^−2^) were recruited and randomly assigned to one of the two groups between 18 March 2022 and 31 December 2022. During the trial period, one patient violated the protocol. As a result, 176 patients (*n* = 88 per group) were included in the ITT analysis and 175 patients were included in the PP analysis (Figure 1). There was no difference between the groups in patient characteristics, including age, height, weight, BMI, ASA physical status and Mallampati score (Table 1).

**Table 1.** Demographic data of the study groups.

Parameters	Fentanyl group (*n* = 88)	Esketamine group (*n* = 87)
Age (years)	30.8 (6.5)	29.2 (6.1)
Height (cm)	160.3 (11.7)	159.3 (5.6)
Body weight (kg)	54.0 (9.0)	52.5 (7.1)
BMI (kg/m^2^)	21.2 (3.6)	20.1 (2.6)
ASA physical status (I/II)	88/0	86/1
Mallampati score (I/II)	65/23	70/17

ASA: American Society of Anesthesiologists; BMI: body mass index.

Data are showed as means (standard deviations) or numbers.

The primary outcomes are summarized in Table 2. The incidence of respiratory rate ≤8 breaths/min in the fentanyl group was significantly higher than that in the esketamine group (24% vs. 6%, *p* = .0011). The incidence of SpO_2_ ≤90% (≥15 s) in the fentanyl group was significantly higher than that in the esketamine group (28% vs. 6%, *p* < .0001). None of the patients in either group developed apnoea. The total incidence of respiratory depression in the fentanyl group was significantly higher than that in the esketamine group (45% vs. 11%, *p* < .0001).

**Table 2.** Incidence of respiratory depression in the study groups.

Outcomes	Fentanyl group (*n* = 88)	Esketamine group (*n* = 87)	Effect size (relative risk) (95% CI)	*p* Value
Patients with respiratory depression	40 (45%)	10 (11%)	3.955 (2.172–7.418)	<.0001
RR ≤8 breaths/min	21 (24%)	5 (6%)	4.152 (1.718 − 10.30)	.0011
Apnoea ≥15 s	0 (0%)	0 (0%)	–	–
SpO_2_ ≤90% (≥15 s)	25 (28%)	5 (6%)	4.943 (2.079 − 12.09)	<.0001

RR: respiratory rate; SpO_2_: pulse oximetry.

Data are presented as numbers (%). Fisher’s exact test was used to analyse differences between groups.

Propofol administration requirement was lower with esketamine than with fentanyl (*p* < .0001). No differences were found in terms of anaesthesia, surgery or recovery times between groups (Table 3).

**Table 3.** Variables of anaesthesia and surgery in the study groups.

Parameters	Fentanyl group (*n* = 88)	Esketamine group (*n* = 87)	Effect size (difference in medians) (95% CI)	*p* Value
Propofol dose (mg)	130 (120–60)	110 (110–140)	−20 (−30 to −10)	<.0001
Anaesthesia time (min)	10 (8.0–12)	10 (8.5–11.5)	0 (−1.0 to 1.0)	.7731
Surgery time (min)	5.0 (4.0–6.0)	4.5 (4.0–6.0)	−0.5 (−1.0 to 0)	.5431
Recovery time (min)	3.0 (2.0–5.0)	4.0 (3.0–5.0)	1 (0–1.0)	.4119

Data are presented as median (IQRs). The differences between groups were analysed by using the Mann–Whitney *U*-test.

**Figure 2.**
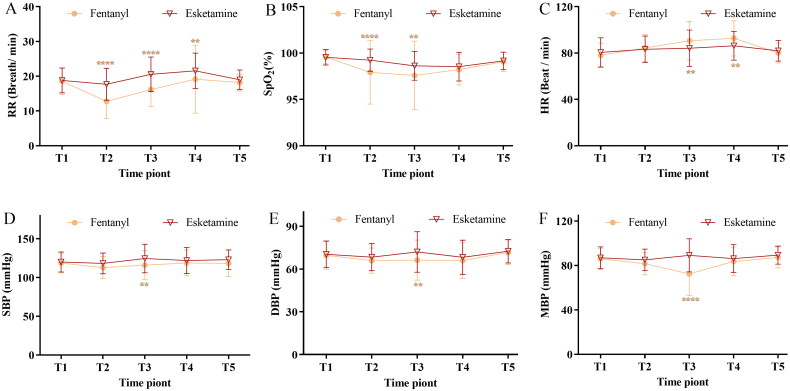
Changes in respiratory rate (A), SpO_2_ (B), heart rate (C), systolic blood pressure (D), diastolic blood pressure (E) and mean blood pressure (F) in patients receiving fentanyl or esketamine for anaesthesia. Data are displayed as means (standard deviations). **p < .01, ****p < .0001, compared with the fentanyl group. Data were compared using repeated-measures two-way analysis of variance with Greenhouse–Geisser correction, followed by Bonferroni’s multiple comparisons test. RR: respiratory rate; SpO_2_: pulse oximetry; HR: heart rate; SBP: systolic blood pressure; DBP: diastolic blood pressure; MBP: mean blood pressure. Time points: T1, 5 min before anaesthesia; T2, at the beginning of surgery; T3, at the end of surgery; T4, at the time of eye opening; and T5, 1 h after surgery.

The vital signs of the patients in the two groups are summarized in Figure 2. The respiratory rate (Figure 2(A)) was higher in the esketamine than in the fentanyl groups at the beginning of surgery (*p* < .0001), end of surgery (*p* < .0001) and time of eye opening (*p* = .0163). The SpO_2_ (Figure 2(B)) was higher in the esketamine than in the fentanyl groups at the beginning of surgery (*p* = .0006) and at the end of surgery (*p* = .0104). Heart rate (Figure 2(C)) was higher in the fentanyl than in the esketamine groups at the end of surgery (*p* = .0058) and at the time of eye opening (*p* = .0055). SBP (Figure 2(D)) was higher in the esketamine than in the fentanyl groups at the end of surgery (*p* = .0110). DBP (Figure 2(E)) and MBP (Figure 2(F)) were higher in the esketamine than in the fentanyl group at the end of surgery (*p* = .0039 and *p* < .0001, respectively).

Adverse events that occurred in both groups are summarized in Table 4. In the esketamine group, the incidences of hypotension (1.1% vs. 11.4%; *p* = .0094), PIP (36.8% vs. 58.0%; *p* = .0064) and the need for chin lifting (5.7% vs. 28.4%; *p* < .0001) were significantly lower than that in the fentanyl group. The incidence of nightmares in the esketamine group was significantly higher than that in the fentanyl group (10.3% vs. 0%, *p* = .0015). No differences were found between the groups in terms of hypertension, tachycardia, bradycardia, physical movement, coughing, nausea/vomiting or pleasant dreams.

**Table 4.** Incidence of adverse event in the study groups.

Parameters	Fentanyl group (*n* = 88)	Esketamine group (*n* = 87)	Effect size (difference in relative risk) (95% CI)	*p* Value
Hypertension	3 (3.4%)	7 (8.0%)	0.5649 (0.1815–1.742)	.3706
Hypotension	10 (11.4%)	1 (1.1%)	9.886 (1.692–59.36)	.0094
Propofol-induced pain	51 (58.0%)	32 (36.8%)	1.576 (1.144–2.206)	.0064
Tachycardia (>100 beats/min)	11 (12.5%)	10 (11.5%)	1.088 (0.4970–2.385)	>.9999
Bradycardia (<50 beats/min)	0 (0%)	1 (1.1%)	0.000 (0.000–3.764)	.4971
Chin lifting	25 (28.4%)	5 (5.7%)	4.943 (2.079–12.09)	<.0001
Physical movement	10 (11.4%)	5 (5.7%)	1.977 (0.7390–5.356)	.2801
Cough	1 (1.1%)	0 (0%)	Infinity (0.2597–infinity)	>.9999
Nausea/vomiting	0 (0%)	2 (2.3%)	0.000 (0.000–1.871)	.2457
Pleasant dream	15 (17.0%)	20 (23.0%)	0.7415 (0.4085–0.337)	.3503
Nightmares	0 (0%)	9 (10.3%)	0.000 (0.000–0.4092)	.0015
Headache	4 (4.5%)	5 (5.7%)	0.6591 (0.205–2.103)	.5355
Dizziness	4 (4.5%)	8 (9.2%)	0.4943 (0.1627–0.485)	.2484

Data are presented as numbers (%). Fisher’s exact test was used to analyse the differences between groups.

## Discussion

To our knowledge, no previous randomized controlled trial has compared the incidence of respiratory depression induced by esketamine and fentanyl in patients undergoing abortion surgery or curettage under propofol-based non-intubated general anaesthesia. Our main finding was that the percentages of patients who developed respiratory depression were lower in the esketamine group than that in the fentanyl group. Vital signs were more stable with esketamine than with fentanyl. The amount of propofol administered and the incidences of hypotension, PIP, and need for chin lifting were significantly lower with esketamine than with fentanyl. However, the incidence of nightmares was significantly higher with esketamine than with fentanyl. Our results confirmed our hypothesis that the percentages of patients who developed respiratory depression with sub-anaesthetic doses of esketamine undergoing abortion surgery or curettage under propofol-based non-intubated general anaesthesia waslower than that with fentanyl.

Currently, propofol and opioids are the cornerstones of intravenous anaesthesia [32–35]. The combination of propofol with fentanyl, which is commonly used for non-intubated general anaesthesia [33–35], may increase respiratory and hemodynamic complications because of their synergistic cardiopulmonary depressant effects. Propofol induces significant respiratory function depression in a dose-dependent manner [36]. Lee et al. reported that the effect-site concentration during spinal anaesthesia with PIRD was 3.8 μg/ml [37]. Taylor et al. reported that anaesthesia induction with propofol 2.5 mg/kg was accompanied by a greater decrease in the respiratory rate and minute volumes [7]. In our study, propofol was injected at a constant rate during anaesthesia induction, and the total dose of propofol remained less than 2.5 mg/kg. We were unable to determine the effect-site concentrations of propofol. However, propofol reportedly decreases the tidal volume by 60% [38] and the inspiration cycle when used at a sedative dose [9], along with possibly decreasing the ventilatory and heart beat responses to hypoxia [39]. We did not record the changes in tidal volume in the present study, but found that the incidence of respiratory depression was lower with esketamine than with fentanyl. This may be because the analgesic effect of esketamine is mainly induced by NMDAR antagonism, which is partly mediated by its action on opioid receptors. Esketamine did not inhibit chemosensory respiratory responses, resulting in no decrease in the tidal volume or rate frequency. In contrast, subanaesthetic doses of esketamine were reported to counter OIRD in young volunteers [40]. The addition of ketamine to propofol provides respiratory protection [41]. Another reason may be that a combination of opioids (e.g. butorphanol and oxymorphone), even at low doses, increases the probability of PIRD during anaesthesia [36].

Vital signs were more stable with esketamine than with fentanyl. In additional, the percentages of patients who developed hypotension were significantly lower with esketamine than with fentanyl. This may be due to the following: first, unlike propofol, which inhibits circulation, esketamine activates the circulation by mildly increasing sympathetic tone. The opposing cardiovascular effects of these drugs appear to be balanced. Second, the administration of propofol was significantly lower in the esketamine than in the fentanyl group, which was consistent with previous reports that esketamine reduces the dose of propofol required during general anaesthesia [34,42]. Cardiorespiratory depression correlated positively with propofol doses.

The recovery time for propofol combined with ketamine remains controversial [34,43,44]. But no difference in the recovery time was found between the groups in our study. The first reason for this may be the different populations recruited. In our study, we enrolled female patients (aged 18–60 years, ASA I–II) who were scheduled to undergo abortion surgery or curettage. Another reason may be the different types and doses of ketamine and esketamine used. Third, different surgical procedures were performed.

In our study, the amount of propofol administered was lower with esketamine than with fentanyl, which was consistent with the findings of previous reports [34]. One benefit of esketamine is that it may reduce the use dose of analgesic and pain in patients with chronic pain[45,46], or in patients undergoing surgery [47,48]. The incidence of PIP with esketamine during anaesthesia induction was lower than that with fentanyl, which was consistent with previous results showing that pretreatment or co-treatment with esketamine prevented PIP [49,50]. Therefore, a combination of esketamine and propofol may decrease the dose of propofol need for anaesthesia, while preserving its sedative and analgesic efficacy.

Subanaesthetic doses of ketamine produced an increase in nightmares [48,51,52]. In our study, the incidence of nightmares in the esketamine group was significantly higher than that in the fentanyl group, but was significantly lower than that in previous studies using ketamine alone [52,53]. The reason for increased nightmares is suspected to be the cognitive alteration induced by esketamine, which can induce a psychotomimetic effect similar to that observed in schizophrenia [54,55]. The analgesic effect and affinity of esketamine for NMDARs are twice those of ketamine. Low-dose esketamine mainly binds to NMDAR in the spinal dorsal horn rather than in the brain, achieving the same analgesia with a lower risk of psychotomimetic adverse effects [47]. In contrast, propofol inhibits these effects by activating GABA receptors. Therefore, the combination of esketamine with propofol may be an appropriate substitute for the combination of fentanyl with propofol in patients undergoing abortion surgery or curettage under non-intubated general anaesthesia, preserving their sedative and analgesic efficacy, while minimizing adverse effects because of their synergistic effect and dose reduction.

Our study had some limitations. First, all patients were recruited from a single centre, which would weaken the external validity of the study. Second, we only detected the effects of a single dose of esketamine (0.15 mg/kg). Lower dose of esketamine may be effective with fewer or no adverse events. Third, due to condition limitations, we did not detect the changes in the tidal volume. Based on the current findings and their limitations, further studies in other populations and using different doses of esketamine are required to confirm our findings.

## Conclusions

This randomized controlled trial found that the incidence of respiratory depression was lower with subanaesthetic doses of esketamine than with fentanyl in women undergoing abortion surgery or curettage under propofol-based non-intubated general anaesthesia. Thus, considering issues of opioid abuse and patient safety, esketamine could be recommended as routine therapy for pain relief in patients undergoing abortion surgery or curettage under non-intubated general anaesthesia.

## Supplementary Material

CONSORT 2010 Checklist.doc

## Data Availability

Data generated in this study are available from the corresponding author upon request.
